# MFBP-UNet: A Network for Pear Leaf Disease Segmentation in Natural Agricultural Environments

**DOI:** 10.3390/plants12183209

**Published:** 2023-09-08

**Authors:** Haoyu Wang, Jie Ding, Sifan He, Cheng Feng, Cheng Zhang, Guohua Fan, Yunzhi Wu, Youhua Zhang

**Affiliations:** 1 School of Information and Computer Science, Anhui Agricultural University, Hefei 230036, China; whycs@stu.ahau.edu.cn (H.W.); dingjie@stu.ahau.edu.cn (J.D.); zhangcheng@stu.ahau.edu.cn (C.Z.); fangh@ahau.edu.cn (G.F.); 2 Anhui Provincial Engineering Laboratory for Beidou Precision Agriculture Information, Anhui Agricultural University, Hefei 230036, China; itsbrqs@stu.ahau.edu.cn (S.H.); fengcheng@stu.ahau.edu.cn (C.F.); 3 School of Natural Science, Anhui Agricultural University, Hefei 230036, China

**Keywords:** pear leaf disease, segmentation model, multi-scale feature extraction, diffusion model, dynamic sparse attention mechanism, MFBP-UNet

## Abstract

The accurate prevention and control of pear tree diseases, especially the precise segmentation of leaf diseases, poses a serious challenge to fruit farmers globally. Given the possibility of disease areas being minute with ambiguous boundaries, accurate segmentation becomes difficult. In this study, we propose a pear leaf disease segmentation model named MFBP-UNet. It is based on the UNet network architecture and integrates a Multi-scale Feature Extraction (MFE) module and a Tokenized Multilayer Perceptron (BATok-MLP) module with dynamic sparse attention. The MFE enhances the extraction of detail and semantic features, while the BATok-MLP successfully fuses regional and global attention, striking an effective balance in the extraction capabilities of both global and local information. Additionally, we pioneered the use of a diffusion model for data augmentation. By integrating and analyzing different augmentation methods, we further improved the model’s training accuracy and robustness. Experimental results reveal that, compared to other segmentation networks, MFBP-UNet shows a significant improvement across all performance metrics. Specifically, MFBP-UNet achieves scores of 86.15%, 93.53%, 90.89%, and 0.922 on MIoU, MP, MPA, and Dice metrics, marking respective improvements of 5.75%, 5.79%, 1.08%, and 0.074 over the UNet model. These results demonstrate the MFBP-UNet model’s superior performance and generalization capabilities in pear leaf disease segmentation and its inherent potential to address analogous challenges in natural environment segmentation tasks.

## 1. Introduction

Pears occupy a significant position among global fruit tree species, favored widely by consumers for their nutrient-rich fruit and distinctive taste [1]. Concurrently, the pear industry’s growth has brought substantial economic benefits to farmers, stimulating the development of local agricultural economies. However, pear trees frequently encounter various diseases during their growth process, which often first manifest on the leaves [2], significantly impacting the tree’s growth and development. These diseases subsequently lead to a reduction in fruit yield and quality, resulting in economic losses for farmers [3].

In traditional agricultural practices, disease detection and identification in pear tree leaves were anchored in experiential knowledge and subjective assessment. Given its inherent limitations in terms of timeliness and precision, this approach falls short of modern agricultural demands. Accurate segmentation of afflicted leaves is paramount for the foundation of a robust plant disease prevention and management framework.

In recent years, machine learning and image recognition technologies have been widely applied across various domains, including healthcare [4], biomimetics [5], food science [6], and information technology [7]. In fact, they have also made significant advancements in agricultural research, enhancing the accuracy and efficiency of plant disease identification [8]. For instance, Poornima et al. [9] employed an image processing technique based on edge and color features for the identification and segmentation of plant disease symptoms, using multi-class Support Vector Machines for disease classification. Ma et al. [10] achieved an impressive accuracy rate of 90.67% in imaging segmentation of greenhouse cucumber downy mildew through a detailed analysis process, employing a decision tree built on a color feature subset selected via Pearson rank correlation. Jothiaruna et al. [11] introduced an innovative disease spot segmentation technique using excessive red index, hue, and a novel color-to-gray conversion algorithm for CCF detection, especially apt for scenarios with uneven illumination or complex backgrounds. This technique even outperformed traditional methods like OTSU [12] and K-means clustering [13].

Although the aforementioned methods perform well under specific conditions [14], their strong reliance on manual feature extraction and selection limits their robustness when dealing with varied and complex morphologies of diseases, potentially leading to a significant decline in segmentation accuracy.

With the advancement of deep learning technologies, significant progress has been made in plant disease identification [15]. Current disease identification approaches can be categorized into two main types: one utilizes bounding boxes to detect specific areas, offering the advantage of rapid disease localization, but may not accurately represent the true extent of the disease. The second is the semantic segmentation approach, which classifies each pixel, thereby distinguishing disease and healthy regions more accurately. While this method is more time-consuming in data annotation, it indeed offers a direct visualization of the actual extent of the disease. As precision and smart agriculture advance, such detailed information becomes increasingly critical, offering precise assessments of disease severity and aiding in implementing effective intervention measures [16]. Tassis et al. [17] introduced a deep learning framework that integrates various convolutional neural networks (Mask R-CNN [18], UNet [19], PSPNet [20], and ResNet [21]) for the automated detection and recognition of in-field coffee tree disease images. This framework achieved an accuracy of 73.90% and a recall of 71.90% in instance segmentation tasks. Patil et al. [22] designed an Enhanced Radial Basis Function Neural Network (ERBFNN) model using the improved MSFO algorithm for tomato leaf disease segmentation. By processing image noise and extracting color features, this model outperformed other methods, achieving an accuracy of 98.92%. Wang et al. [23] introduced a strategy for classifying the severity of cucumber leaf diseases, combining DeepLabV3+ [24] and U-Net. Compared to other methods, this model demonstrated superior robustness, segmentation precision, and classification accuracy, with an average accuracy of 92.85%. Zhang et al. [25] proposed an enhanced UNet method (MU-Net), incorporating residual blocks and paths. This strategy effectively addressed the gradient vanishing and exploding problems of U-Net and strengthened feature information transfer. Experimental results indicated improved accuracy and efficiency in plant diseased leaf image segmentation.

Despite the encouraging performance exhibited by various advanced models in plant disease identification research, significant challenges remain. Existing models frequently underperform in segmentation tasks, especially when the disease features are minute, undergo substantial morphological variations, or present with indistinct boundaries [26]. Addressing these challenges, we first employ multi-scale and multi-type convolutional kernels to capture features at different scales, allowing better adaptability to disease variations under different conditions. Balancing local and global information has always been a challenging issue for existing network models. Hence, we introduce dynamic sparse attention mechanisms and tokenized multi-layer perceptron modules. This not only aids in precisely extracting lesion edge information but also ensures the model’s robustness in complex scenarios. The primary contributions of this paper can be summarized in three respects:

1. We devised and implemented an innovative Multi-Scale Feature Extraction (MFE) module. This system leverages multi-scale, multi-type convolutional kernels, frequency attention mechanisms, and residual connections to enhance the model’s ability in complex image feature recognition and generalization.
2. We introduced the Tokenized Multilayer Perceptron (BATok-MLP) module, which operates on the basis of dynamic sparse attention. By integrating region-level attention with global attention, this module effectively balances the model’s proficiency in both global and local information extraction.
3. We pioneered the application of a diffusion model in data augmentation tasks for pear leaf disease segmentation. In addition to performing standalone tests for various data augmentation techniques, we have conducted a comprehensive analysis of the combined effect of different enhancement strategies.

The remainder of this paper is organized as follows: Section 2 introduces the acquisition of the dataset and describes the proposed MFBP-UNet method, Section 3 presents and discusses the experimental results, and Section 4 delves deeper into a discussion of the results. Finally, Section 5 concludes the paper.

## 2. Materials and Methods

### 2.1. Data Acquisition

**Figure 1:**
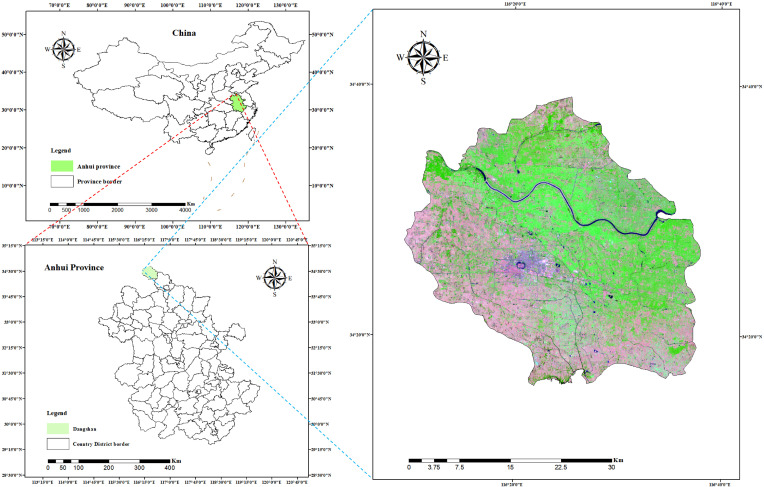
Location of dataset sources.

The pear leaf disease image dataset utilized in this study was mainly derived from two sources. To begin, a significant portion of our data was directly collected from a pear orchard located in Dangshan County, Suzhou City, Anhui Province. This collection was facilitated by the expert guidance of local arborists (Dangshan County’s geographical location is shown in Figure 1). This collection utilized a Canon 700D camera with a 5184 × 3456 pixels resolution, maintaining a shooting distance of 10 cm to 20 cm. To ensure diversity in lighting conditions, images were taken during various times of day and weather conditions. Through this process, we obtained images of a range of pear leaf diseases, including 838 instances of Rust, 213 instances of Slug, and 57 instances of Curl.

**Figure 2:**
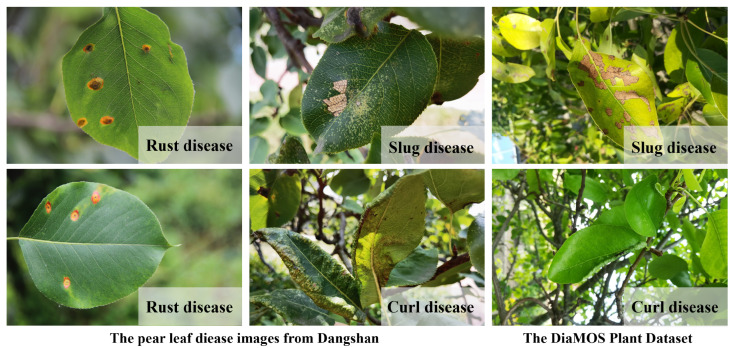
Display of disease images from different dataset sources.

To enhance our dataset, we opted to employ the DiaMOS Plant Dataset [27], a public plant disease image dataset. Although the pear leaf diseases in this dataset can be segregated into three categories, due to the unbalanced distribution of labels, we decided to only select images from two disease categories for further analysis; Figure 2 displays partial pear leaf disease images from different data sources.

After conducting rigorous screening and processing, we curated a final set of 1374 high-quality images of pear leaf disease for the experimental phase of this research. The selection process for these images was meticulously controlled to ensure the diversity and efficacy of the experiments. Consequently, this laid a solid and expansive data foundation for the following stages of our investigation.

### 2.2. Dataset Processing and Enhancement

**Figure 3:**
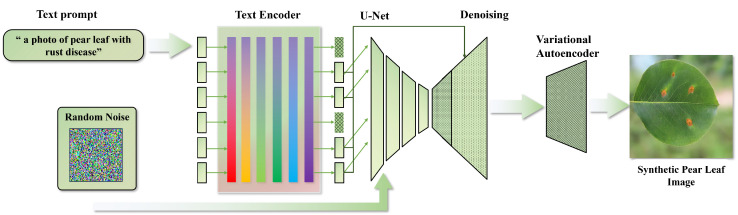
The diffusion model pipeline for synthetic pear leaf disease image generation.

To address the issue of data scarcity and to further augment the generalization performance of our model, we innovatively introduced a diffusion model, in conjunction with data augmentation techniques, to enhance the diversity of our dataset. Our approach incorporated a tripartite diffusion model for stable pear leaf disease progression, illustrated in Figure 3.

1. Text Encoding: Utilizing the CLIP model [28], each token from the input text prompt, which describes the pear leaf disease, is transformed into an embedding vector.
2. Latent Space U-Net Generator: This component accepts all the token embeddings and an array of random noise, sequentially generating an array of elements that more accurately reflects both the input text and the images of pear leaf disease, which the U-Net has been trained on.
3. Image Decoder: Employing a Variational AutoEncoder (VAE), the acquired latent arrays are converted into pixel-space representations of pear leaf diseases. Throughout this workflow, the embedded vectors, derived from the text encoding that describes the pear leaf disease, regulate the generation of latent space representations by the U-Net and the decoding process of the VAE.

We utilized a variety of concrete data augmentation strategies:

1. Random rotation: Involving the rotation of images at arbitrary angles to enhance the spatial distribution of the data.
2. Random color dithering: Where random adjustments are made to the image’s saturation, contrast, brightness, and sharpness to simulate disease features in varying conditions.
3. Adding Gaussian noise: To emulate diverse disease characteristics and bolster the model’s robustness against noise.
4. Mirror flipping: Encompassing both vertical and horizontal flipping, contributing to an improved spatial distribution of the data.
5. Stable diffusion: To combat sample scarcity and further improve the generalization of the model.

**Figure 4:**
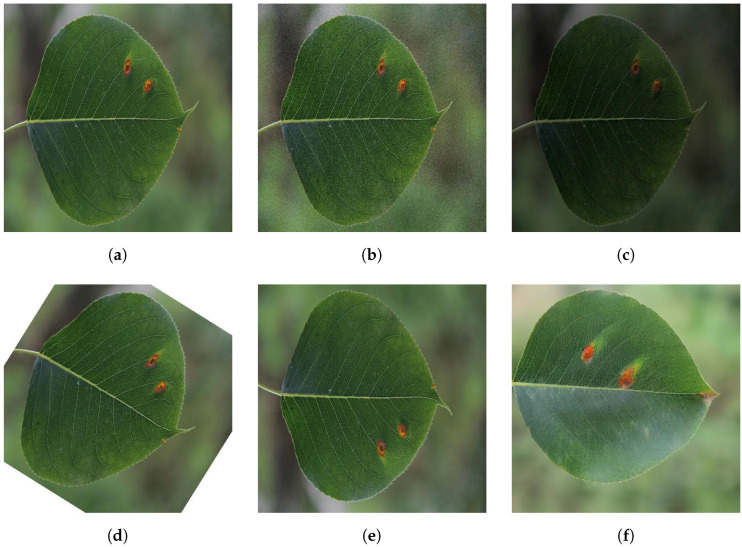
Examples of different data enhancement methods. (**a**) Original; (**b**) Adding Gaussian noise; (**c**) Random color dithering; (**d**) Random rotation; (**e**) Mirror flipping; (**f**) Stable diffusion.

Figure 4 displays images of pear leaf diseases following data augmentation. These augmentation techniques enable the model to carry out more effective pear leaf disease segmentation under complex conditions. Concurrently, Table 1 provides a breakdown and count of the enhanced datasets of the three types of pear leaf diseases that we gathered.

**Figure 5:**
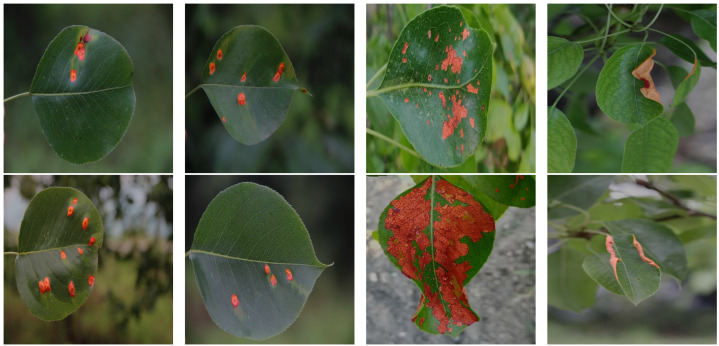
Presentation of some sample labels.

To efficiently train our segmentation model, we employed the Labelme software to meticulously annotate the diseased portions of the pear leaves, resulting in the creation of corresponding mask images. These mask images (as shown in Figure 5) serve as precise training targets for the model, thereby amplifying the effectiveness of model learning. The annotation information for each image is stored in the Json format, facilitating easy reuse and sharing of these annotations.

### 2.3. Methods

#### 2.3.1. MFBP-UNet Overall Architecture

**Figure 6:**
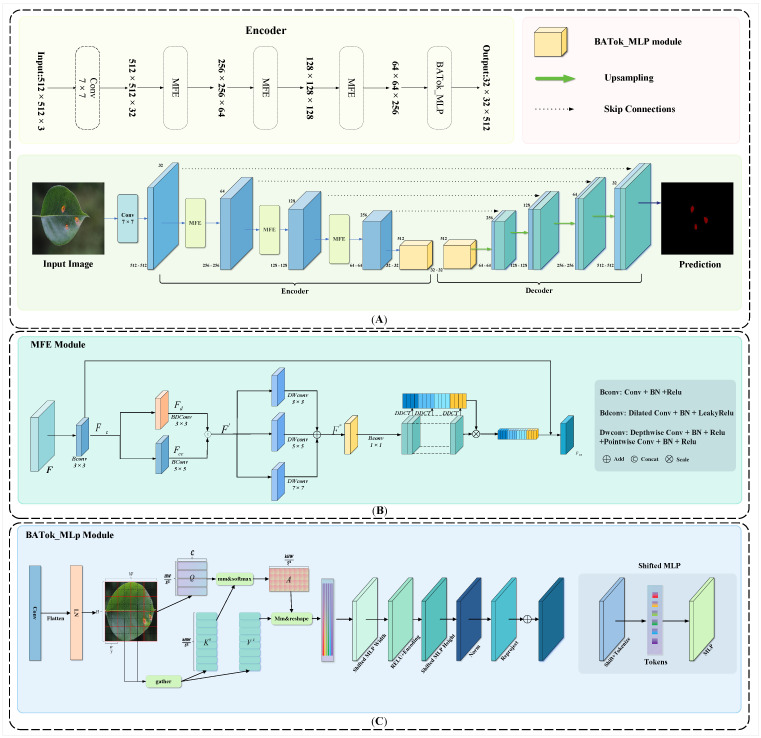
MFBP-UNet structure diagram. (**A**) The overall structure of MFBP-UNet. (**B**) Architecture of MFE. (**C**) Architecture of BATok-MLP.

In our study, mindful of the impact of complex backgrounds and disparate lighting conditions, we developed a multi-disease segmentation network termed MFBP-UNet, which is based on the UNet architecture.The overall network architecture is shown in Figure 6. The novelty of this network lies in its two key feature extraction modules: the MFE module and the BATok-MLP module. The MFE module fully leverages multi-scale and multi-type convolution kernels to effectively extract detailed and semantic features. To bypass traditional pooling operations, we specifically integrated a frequency attention mechanism to enhance the model’s robustness and amplify its disease feature recognition capability. In the same vein, the BATok-MLP module, while curbing model complexity, successfully incorporates a dynamic sparse attention mechanism, enabling the effective utilization of global information and the attainment of a dynamic trade-off between global and local features. In terms of optimization, we adopted a combination of cross-entropy loss and Dice loss methods. This amalgamation empowers the model to effectively recognize and pinpoint pear leaf disease spots in complex environments, especially as the Dice loss method can address class imbalance issues more competently. The specific computation formula is as follows:

(1) $$L_{CE}=-\sum_{i=1}^{m}t\times \log\left(y\right)$$

(2) $$L_{\mathrm{Dice}\;}=1-\frac{2\sum_{i=1}^{n}y\times t+\varepsilon }{\sum_{i=1}^{n}y+\sum_{i=1}^{n}t+\varepsilon },$$

where *t* stands for the true labels while *y* signifies the model’s predicted output. *m* denotes the number of classes, *n* denotes the number of pixels, and ε is a hyperparameter preset to $1\times e^{-5}$, serving as a safeguard against zero denominator scenarios.

(3) $$L=\alpha \times L_{CE}+\beta \times L_{\mathrm{Dice}}.$$

In this formula, α and β serve as weight coefficients and are respectively configured to 1 and 0.5.

Our research has elucidated that these amendments noticeably bolster the overall performance and precision of our model in comparison to UNet and other advanced networks. The primary driving force behind this enhancement is the introduction of innovative feature extraction modules and optimization strategies. These implementations ensure the model’s proficient recognition and localization capabilities under the challenges posed by complex backgrounds and diverse lighting conditions.

#### 2.3.2. MFE Module

The convolution operation within Convolutional Neural Networks plays a critical role in local feature extraction from images, efficiently encapsulating detailed aspects of the image while preserving spatial information. Nonetheless, Convolutional Neural Networks tend to rely on pooling operations as a means to reduce computational complexity and curb overfitting. This approach, however, can compromise the model’s sensitivity to smaller targets during the dimensionality reduction process. To address this issue and ensure a more precise detection and localization of pear leaf disease within intricate environments, we devised a multiscale type module that incorporates residual connections. As opposed to conventional Convolutional Neural Networks, this module circumvents the need for pooling operations. Instead, it harnesses detail-rich and semantic features by operating at different convolution scales and levels, thereby more effectively preserving the continuity of information. Figure 6B shows its architecture.

The operation of the Multiscale Feature Extraction (MFE) module is as follows: First, a 3 × 3 convolution layer is used to extract features from the input feature map $F\in R^{B\times H\times W\times C}$, producing a base feature map $F_{c}$. This basic feature map $F_{c}$ is then fed into two separate branches: one uses a 3 × 3 extended convolution to extract a detail-oriented feature map $F_{d}$, rich in local details; the other uses a 5 × 5 convolution layer to extract a more global, semantic-oriented feature map $F_{cc}$. The resulting feature maps $F_{d}$, $F_{cc}$ from these two branches are then merged along the channel dimension to produce the final feature map $F^{\prime }$.

(4) $$F^{\prime }=Concat\left(F_{d},F_{cc}\right),$$

where Concat denotes the operation of concatenation.

Upon this novel feature map, we implemented depthwise convolution operations with kernels of sizes 3×3, 5×5, and 7×7. This particular operation significantly mitigates computational complexity and the volume of model parameters while concurrently sustaining the potent feature extraction capacity. By adopting convolution operations of diverse scales, we could extract features of varying dimensions, in turn bolstering the model’s expressivity. We then integrated the outputs across these three branches, thus furnishing a richer and more comprehensive representation encapsulating expansive disease feature information.

(5) $$F^{\prime \prime }=\phi_{1}\left(D_{3}\left(F^{\prime }\right)+D_{5}\left(F^{\prime }\right)+D_{7}\left(F^{\prime }\right)\right),$$

where $\phi_{1}$ signifies a 1 × 1 convolutional layer designed for channel compression and dimensionality reduction. This operation simultaneously merges features of differing scales, leading to a reduction in the feature map’s dimensionality and an enhancement of the model’s efficiency. $D_{i}$ symbolizes an i×i(i=3,5,7) dilated convolution layer.

Finally, a frequency attention mechanism was incorporated to bolster the model’s focus on significant frequency features within the image. This mechanism fortifies the model’s comprehension of global information by amplifying its capacity to detect disease-related features, subsequently increasing model robustness. Concurrently, the frequency attention mechanism is effective in mitigating the effects of superfluous information and noise—such as random textures and background noise prevalent in the image—thereby elevating the precision and reliability of the model’s segmentation of pear leaf disease.

In practical applications, the initial step involves partitioning the input feature map into several groups, each subjected to a two-dimensional discrete cosine transform (2DDCT). Post-processing via a fully connected layer and function yields the ultimate weighted feature map. Subsequently, we employ a residual structure to integrate this weighted feature map with the foundational feature map, thus producing the final output feature map.

(6) $$F^{\prime \prime }=\left[F^{\prime \prime }0,F^{\prime \prime 1},\ldots ,F^{\prime \prime n-1}\right]$$

(7) $$Freq=\mathrm{Concat}\left(\left[2DDCT\left(F^{\prime \prime }\right),2DDCT\left(F^{\prime \prime 1}\right),\ldots ,2DDCT\left(F^{\prime \prime n-1}\right)\right]\right)$$

(8) $$F_{\mathrm{out}\;}=\mathrm{Sigmoid}\left(FC\left(2DDCT\left(F^{\prime \prime }\right)\right)\right)\times F^{\prime \prime }+\phi_{3}\left(F\right),$$

where Sigmoid signifies the activation function, FC stands for the fully connected network layer, Freq embodies the frequency domain features of the input, which is obtained through 2DDCT transformation, and n is a predetermined value denoting the subdivisions of the input feature.

Within the MFE module, we harnessed the power of a variety of convolution operations of different types and scales for feature extraction, thereby amplifying the expressive power of the features. Subsequently, we incorporated a frequency attention mechanism, which enhances the model’s focus on significant frequency features of the image by adjusting the features in the frequency domain, resulting in improved robustness and accuracy of the model. Such a design equips the module with the ability to efficiently detect and precisely locate pear leaf disease even in complex environments.

#### 2.3.3. BATok-MLP Module

The architecture of UNet is grounded in an encoder-decoder scheme, where a succession of convolutional and deconvolutional operations are employed for progressive encoding and decoding, extracting and mapping features across diverse hierarchical levels. Lower levels primarily focus on fine-grained features (such as edges and textures), while higher levels concentrate on more abstract and global features (like objects and scenes). However, traditional convolutional approaches often lack the capacity to fully comprehend global contextual information, which may lead to the loss of fine-grained features. To address this issue, we designed a novel BATok-MLP module that incorporates a dynamic sparse attention mechanism, as shown in Figure 6C. This module can more effectively process global contextual information, thereby minimizing the loss of fine-grained features.

The Tok-MLP [29] module effectively captures fine-grained features by moving across the width and height of the feature map, allowing the model to focus on specific positions of the convolutional features. However, its extraction of global information is still limited, as the collection of global information primarily relies on the accumulation of local attention across various regions.

As a result, we improved the Tok-MLP module by introducing a dynamic sparse attention [30] mechanism. This mechanism can select different parts of the input sequence for focus in each computation, allowing the model to understand information from a global perspective after multiple iterations. This dynamic attention adjustment can both capture global information and maintain sensitivity to local details.

In the BATok-MLP module, we also introduced a PatchEmbedding layer. This layer includes a 2D convolutional layer and a layer normalization operation, which can transform 2D features into 1D sequential features while preserving spatial information. This transformation allows the model to handle 2D spatial information within a 1D sequence, thus better integrating feature information and enhancing the model’s feature extraction capabilities.

The working principle of the BATok-MLP module can be divided into the following steps: First, we apply the PatchEmbedding layer to process the input feature map $X\in R^{B\times H\times W\times C}$, and then reshape the output one-dimensional sequence $E_{p}$ into $X_{reshaped}\in R^{B\times H\times W\times C}$ to meet the requirements of subsequent operations. The specific operations are as follows:

(9) $$X_{t}={\left(Flatten\left(Conv\left(X,K\right)\right)\right)}^{T}$$

(10) $$E_{p}=\left(X_{t}-\mu \left(X_{t},dim=1\right)/\sqrt{\left(\sigma^{2}(X_{t},dim=1\right)+\varepsilon )}\right),$$

where Conv() represents convolutional operations, μ() and σ() denote mean and variance, respectively, corresponding to the feature dimension. ε is a hyperparameter, set as $\;1\times e^{-6}$. The Flatten() operation converts two-dimensional features into one-dimensional sequential features, and $E_{p}\in R^{B\times N\times C}\left(N=H\times W\right)$ is the output one-dimensional embedded features.

Subsequently, we divide the reshaped input feature map $X_{reshaped}$ into multiple non-overlapping regions of size S×S, and each region is integrated into a feature vector. Then, we perform linear projections on each feature vector to obtain three tensors: *Q*, *K*, and *V*.

(11) $$F_{r}=\;Partition\left(X_{reshaped},\;S\right)$$

(12) $$Q=F_{r}\cdot W_{q},K=F_{r}\cdot W_{k},V=F_{r}\cdot W_{v},$$

where $W_{q}$, $W_{k}$, and $W_{v}$ are learnable weight matrices.

Upon acquiring the tensors of queries (*Q*), keys (*K*), and values (*V*), we calculate the attention scores among regions. Initially, we compute the mean of *Q* and *K* on a specific dimension (dim=1), to obtain region-level queries and keys ($Q_{r}$ and $K_{r}$). Then, we perform a matrix multiplication of $Q_{r}$ and $K_{r}$ to generate the adjacency matrix $X_{r}$, reflecting the correlation degree among regions. Following this, we execute a top−k operation, gathering indices of the *k* most relevant regions from each region, denoted as $I_{r}$. For each region *i*, we use $I_{r}$ to collect the most relevant key-value pairs from *K* and *V*, denoted as $K_{g}$ and $V_{g}$. Ultimately, we perform an attention operation on the collected key-value pairs to yield the output $X^{\prime }$. The corresponding mathematical expressions are as follows:

(13) $$Q_{r}=\mu \left(Q,\dim=1\right),K_{r}=\mu \left(K,\dim=1\right)$$

(14) $$X_{r}=Q_{r}{\left(K_{r}\right)}^{T}$$

(15) $$I_{r}=\mathrm{Top}K\left(X_{r},k,\;\mathrm{axis}\;=1\right)$$

(16) $$K_{g}=\mathrm{Gather}\left(K,I_{r}\right),\;V_{g}=\mathrm{Gather}\left(V,I_{r}\right)$$

(17) $$X^{\prime }=\mathrm{Attention}\left(Q,K_{g},V_{g}\right),$$

where $Q_{r}$ and $K_{r}$ are region-level queries and keys; $X_{r}$ denotes the adjacency matrix representing the correlation degree among regions; $I_{r}$ is the indices of the *k* most relevant regions for each region; $K_{g}$ and $V_{g}$ are the most relevant key-value pairs; and $X^{\prime }$ is the output obtained after the attention operation on the collected key-value pairs.

After the dynamic sparse attention mechanism completes information extraction, we reshape the attention map $X^{\prime }$ into $X^{\prime }\in R^{B\times N\times C}$ and input it into the TokMLP module. In this module, we first perform a shift operation on the width dimension, then utilize a 3×3 convolution kernel, and convert the channel number into the embedding dimension *E* to achieve feature tokenization. Subsequently, these tokens are processed through a ShiftedMLP with a hidden layer dimension of *H* and further processed through a depthwise convolution layer. The corresponding mathematical expressions are as follows:

(18) $$X_{shifted}\;=\;Shift_{width}\left(X\right)$$

(19) $$T_{W}\;=\;Tokenize\left(X_{shifted},\;kernel_{size}=3,\;channels=E\right)$$

(20) $$Y\;=\;DWConv\left(MLP\left(T_{W},\;hidden_{dim}=H\right)\right),$$

where *W* denotes the width of the feature map and DWConv represents a depthwise separable convolution.

During the processing stage after depthwise convolution, we first apply the ReLU [31] activation function to enhance the nonlinear representation ability of the feature map *Y*, obtaining the activated feature map $O^{\prime }$. Then, we perform a shift operation on the height dimension to generate $Y_{shifted}$. Next, using a 3 × 3 convolution kernel and setting the channel number to the embedding dimension *E*, we tokenize these shifted features for the second time, forming tokens $T_{H}$. These newly formed tokens $T_{H}$ are sent to another ShiftedMLP module for processing. This ShiftedMLP module has an output dimension of *O* and its output is denoted as *Z*. Importantly, to introduce long-range dependency of features, we add a residual connection here, that is, the original tokens $T_{H}$ are added to *Z*.

Finally, layer normalization is performed on the features to ensure the stability of feature distribution among layers, which benefits model training and generalization. We denote the output of this step as $Y_{final}$. The mathematical expressions for this series of operations are as follows:

(21) $$Y_{shifted}\;=\;Shift_{width}\left(Y\right)$$

(22) $$T_{H}\;=\;Tokenize\left(Y_{shifted},\;kernel_{size}=3,\;channels=E\right)$$

(23) $$Z=MLP\left(\mathrm{ReLU}\left(T_{H}\right),\;{\mathrm{output}}_{\dim}=O\right)$$

(24) $$Y_{final}\;=\;LN\left(Z\;+\;T_{H}\right),$$

where $Shifted_{height}$ represents a shift operation along the height dimension, ReLU denotes the ReLU activation function, Tokenize stands for tokenization, MLP refers to MLP with specific output dimensions, and LN represents layer normalization.

We inserted the BATok-MLP module at the end of the feature extraction stage of the UNet network encoder, which effectively integrates local and global information. Within the Tok-MLP module, we enhance the capture of fine-grained features by moving the width and height of the features. Additionally, we address the weakness of traditional convolutional methods in understanding global contextual information through dynamic routing sparse attention mechanism, significantly improving the model’s understanding and representation capabilities.

## 3. Results

### 3.1. Experiment Setting and Training Details

To ensure fair comparison of the experiments, we conducted all experiments under a unified hardware and software environment. The network model was built on the PyTorch framework, and details of the relevant hardware and software configurations can be found in Table 2.

### 3.2. Training Setting

The size of the images is uniformly adjusted to 512 × 512 pixels for research purposes. Through the application of our finalized augmentation strategy, we expanded the original dataset to a grand total of 4773 images. To effectively evaluate the model performance and prevent overfitting, we employed 10-fold cross-validation. Under this strategy, all images were randomly assigned to training, validation, and test sets, which accounted for 80% (3819 images), 10% (477 images), and 10% (477 images) of the total dataset, respectively.

To prevent the model from getting stuck in local optima, we used the cosine annealing strategy during training, which helped accelerate model convergence and improve training stability. The entire training process consisted of 100 epochs, which was determined based on our preliminary experimental results to strike the best balance between efficiency and accuracy. The learning rate was dynamically adjusted between $1\times 10^{-4}$ and $1\times 10^{-6}$ to adapt to the model’s needs at different stages of training. We chose Adam with a momentum of 0.9 as the optimizer because it combines the advantages of RMSProp and Momentum optimization strategies, which can reduce gradient oscillation and accelerate model convergence. To optimize computational efficiency and training speed, we set the batch size to 8. Detailed experimental configurations can be found in Table 3.

### 3.3. Evaluation Indicators

The present study employs a comprehensive set of metrics, including Mean Intersection over Union (MIoU) [32], Mean Precision (MPrecision), Mean Pixel Accuracy (MPA), Dice Coefficient [33], Frames per Second (FPS) [34], and Parameters to evaluate model performance comprehensively.

Mean Intersection over Union (MIoU) is a critical metric for evaluating image segmentation performance, which represents the average ratio of the intersection to the union of predicted results and true labels. The calculation formula is as follows:

(25) $$MIoU=\frac{1}{n+1}\sum_{i=0}^{n}\frac{p_{ii}}{\sum_{j=0}^{n}p_{ij}+\sum_{j=0}^{n}p_{ji}-p_{ii}},$$

where *n* represents the number of disease categories, $p_{ii}$ denotes the pixel count where the *i* disease category is correctly predicted as *i* category, and $p_{ij}$ and $p_{ji}$ represent the pixel count, where the *i* and *j* disease categories are mistakenly predicted as each other, respectively.

Mean Precision (MPrecision) and Mean Pixel Accuracy (MPA) are two fundamental evaluation metrics. They represent the proportion of actually positive samples among the samples predicted as positive, and the proportion of samples that are actually positive and are predicted as positive, respectively. The formulas for calculating these two indicators are as follows:

(26) $$MPrecision=\frac{\sum_{1}^{n}\frac{TP}{TP+FP}}{n}$$

(27) $$MPA=\frac{1}{n+1}\sum_{i=0}^{n}\frac{p_{ii}}{\sum_{j=0}^{n}p_{ij}},$$

where TP denotes True Positives while FP denotes False Positives, and *n* represents the number of disease categories detected, $p_{ij}$ represents the number of pixels where category *i* is predicted to be category *j*, $p_{ii}$ denotes the number of pixels correctly predicted to be category *i*.

The Dice Coefficient (also known as the Sørensen–Dice coefficient) is used to measure the similarity between two samples. The formula is as follows:

(28) $$\mathrm{Dice}\;=\frac{2*TP\;}{2*TP+FP+FN},$$

where FN denotes False Negatives.

Frames Per Second (FPS) is a key metric for the detection speed of the model, representing the number of images processed per second. The calculation formula is as follows:

(29) $$FPS=\frac{1}{t},$$

where *t* represents the time taken to process a single image.

### 3.4. Experimental Results and Analysis

#### 3.4.1. Comparison of Different Data Augmentation Methods

**Figure 7:**
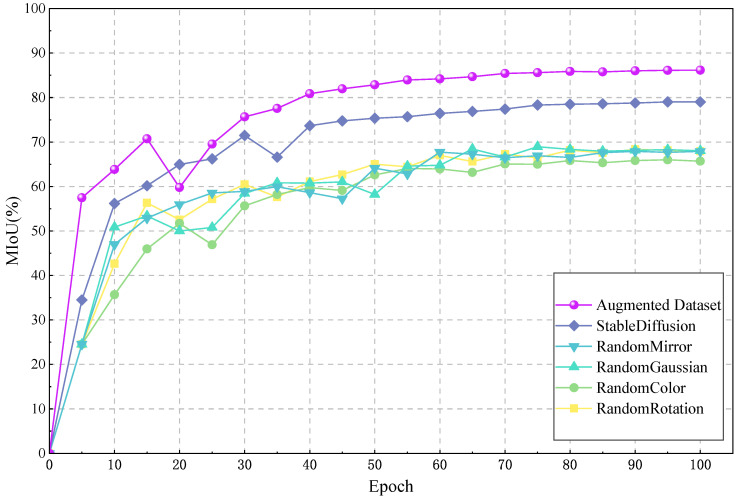
Comparison experiments of different data augmentation effects.

In this study, we systematically investigated the impact of data augmentation on improving the accuracy of pear tree leaf disease segmentation. As shown in Figure 7, we conducted independent experiments for different enhancement methods and deeply explored the effect of combining various enhancement methods. The specific experimental settings were as follows: one group of the original dataset was not subjected to any data augmentation, while five groups of experiments each used random rotation, random color dithering, Gaussian noise addition, mirror flipping, and stable diffusion augmentation methods. The final group of experiments combined all augmentation methods.

The experimental results indicate that the model trained on the original dataset has an accuracy of 65.68%, while the model trained using a single augmentation method has an accuracy ranging from 67.88% to 76.22%. Of particular note is that the stable diffusion method provides the highest model accuracy, reaching 79.01%. This strongly demonstrates that data augmentation can effectively improve model performance by providing neural networks with richer opportunities for feature learning. The introduction of the stable diffusion model in data augmentation for pear tree leaf disease is a first, and the experimental results fully demonstrate its outstanding effectiveness in alleviating the problems of imbalanced dataset and difficult data acquisition.

Although methods such as random rotation, adding Gaussian noise, and random mirroring can change the perspective or contrast of images, their effect on improving model accuracy is not significant. This may be because these methods do not alter the essential features of the plant disease. On the contrary, a comprehensive enhancement method, which combines all data augmentation strategies, improved the model accuracy to 86.16%. This result further confirms that by comprehensively utilizing various enhancement methods, we can train the model from multiple perspectives, improve its generalization ability, and better handle new, unseen data.

In summary, the experimental results strongly support the important role of data augmentation in improving model performance. Particularly, the comprehensive data augmentation method demonstrates significant advantages in enhancing model generalization ability and accuracy. After careful consideration, we selected five methods for our final data augmentation strategy: random rotation, mirror flipping, random color dithering, stable diffusion, and adding Gaussian noise, with a ratio of 1:1:1:2:2.

#### 3.4.2. Experiment Comparing with Different Models

**Figure 8:**
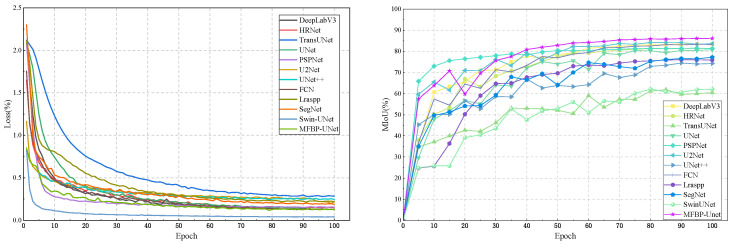
Loss and MIoU curves of the MFBP-UNet and other methods.

In this study, we conducted an in-depth analysis of the performance of the MFBP-UNet model and compared it with numerous widely used image segmentation models. Specifically, we compared it with various well-known UNet-based architectures, such as UNet, UNet++ [35], and U2Net [36], as well as recent transformer-based variants, such as TransUNet [37] and SwinUNet [38]. Additionally, the evaluation process included baseline networks such as FCN [39], DeepLabV3 [40], HRNet [41], PSPNet, Lraspp [42], and SegNet [43]. The experimental results showed that the MFBP-UNet outperformed all the compared models in multiple performance metrics, including MIoU, MPrecision, MPA, and Dice coefficient. Detailed results and training process can be found in Table 4 and Figure 8.

Specifically, MFBP-UNet achieved 86.15% MIoU, which is significantly better than all other models, such as DeepLabV3, HRNet, PSPNet, and FCN, with MIoU scores of 83.39%, 81.28%, 82.06%, and 83.21%, respectively. As the key indicator for evaluating model performance in terms of classification accuracy, the superior performance of our model is mainly attributed to the frequency attention mechanism introduced by the MFE module, as well as the design of multi-scale and multi-type convolution modules, which effectively capture enhanced details and semantic features. Meanwhile, the dynamic sparse attention mechanism introduced by the BATok-MLP module enables the model to capture global information while remaining sensitive to local details.

According to the results, MFBP-UNet outperformed all other models in terms of mean pixel accuracy (MPA), achieving an impressive 90.89%. In contrast, Lraspp, U2Net, and UNet++ scored 85.57%, 89.65%, and 89.85% respectively. MPA is a reliable indicator of how accurately a model predicts pixel class, and this outcome further confirms our model’s exceptional performance in extracting details and obtaining semantic information. In the experiment of the Dice Similarity Coefficient (DSC), the MFBP-UNet model achieved a high score of 92.18%, thanks to its ability to accurately locate and identify the boundaries of pear leaf diseases. This experimental result confirms the strong performance of the MFBP-UNet model in handling complex plant disease image segmentation tasks. Additionally, the result also indicates that our model performs well in extracting and fusing features at a global scale, as well as obtaining details and boundary information, which is crucial in image segmentation tasks.

However, other models, such as SwinUNet and TransUNet, have shown a relatively weaker performance on these metrics. For instance, TransUNet’s MIoU and Dice coefficients are 62.47% and 0.828, respectively, which are significantly lower than our model’s. The reason for this could be that their structural design overlooks the importance of multi-scale features and has a large number of parameters, leading to significant hardware requirements for leaf disease image segmentation tasks.

Finally, we report the average inference time when running on a CPU. Considering that most edge devices operate on low computing power and often lack the computational advantage of GPUs, we performed forward propagation on ten 512 × 512 pixels resolution images and reported the average inference time. Although MFBP-UNet did not perform optimally in the FPS metric, its speed of 4.603 is still acceptable. It is worth noting that the parameter count of MFBP-UNet is 22.076297M, much lower than that of models such as TransUNet and SwinUNet. This allows MFBP-UNet to efficiently utilize computing resources when handling large-scale and complex tasks, and can also be applied effectively on hardware-limited devices.

#### 3.4.3. Ablation Experiment

**Figure 9:**
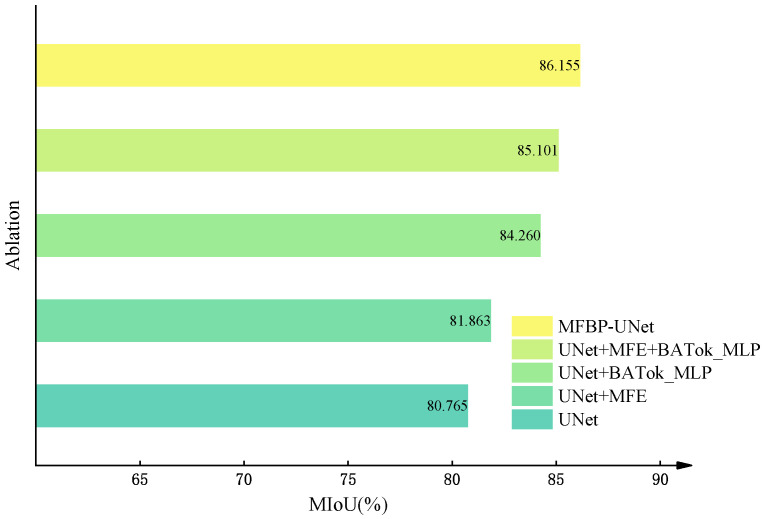
Ablation experiments of MFBP-UNet.

**Figure 10:**
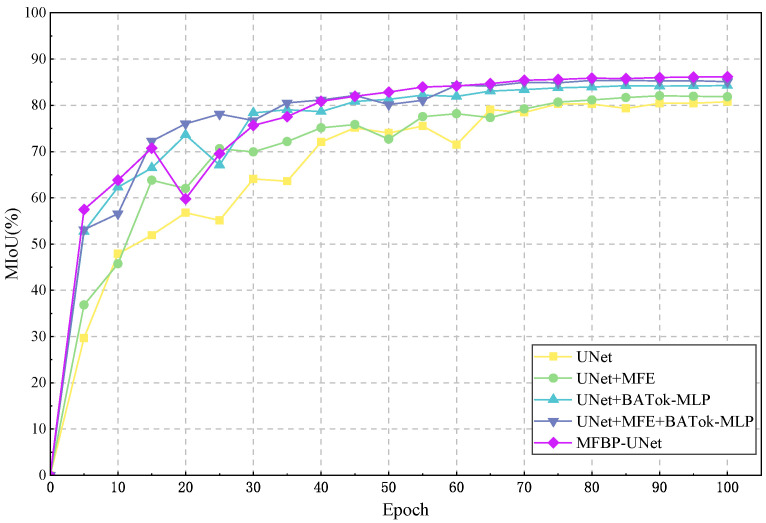
Experimental results of ablation experiment effects.

To demonstrate the performance improvement resulting from a series of improvements made to the UNet network model, we trained and tested on an enhanced dataset, with MIoU selected as the primary evaluation metric. Using the controlled variable method, we introduced the multi-scale feature fusion (MFE) module and the BATok-MLP module one by one, and combined the cross-entropy loss and Dice loss functions to conduct a series of ablation experiments. Figure 9 shows a comparison of performance evaluation indicators under different module configurations, including no module enhancement, using only the MFE module, using only the BATok-MLP module, using the MFE and BATok-MLP modules in combination, and the optimized MFBP-UNet model using cross-entropy and Dice loss functions. Meanwhile, Figure 10 details the MIoU variation curves of each ablation experiment on the training set.

After embedding the MFE module, we observed a 1.1% increase in the model’s MIoU. This improvement can be mainly attributed to the MFE module’s ability to extract and fuse feature information from multiple scales. In this way, the module enhances the network’s understanding of global contextual information while also improving its sensitivity to subtle local variations. As a result, the model’s ability to recognize small targets is enhanced.

Subsequently, we integrated the BATok-MLP module. By employing this module and leveraging a dynamic sparse attention mechanism, we enhanced the model’s capacity to process global and local information, achieving a dynamic equilibrium of features and thereby improving the model’s performance. This strategy mitigates the model’s reliance on non-essential information, reinforces the extraction of key information, effectively reducing redundant data within the model and further fortifying its representational ability.

Finally, we replaced the loss function of the network with a combination of cross-entropy loss and Dice loss. The cross-entropy loss function helps the model to recognize each category evenly, while the Dice loss function has good robustness for imbalanced samples, especially for minority classes (small targets), significantly improving the recognition ability. The design of this loss function enhances the model’s attention to small targets, further improving the model’s performance.

Overall, our improvement strategy, including the introduction of the MFE module and BATok-MLP module, as well as the optimization of the loss function, successfully increased MIoU by 5.4% while keeping the parameter volume relatively small. This result fully validates the effectiveness and rationality of our improvement strategy.

### 3.5. Prediction Result Display of MFBP-UNet

As illustrated in Table 5, we demonstrate the disease segmentation results of 12 models on the test set. Different diseases, such as Rust, Curl, and Slug, are represented in red, yellow, and green, respectively, while the background is denoted in black. Preliminary observation reveals that some benchmark models like PSPNet, Lraspp, UNet++, and SegNet have significant shortcomings in handling pear leaf disease segmentation tasks; particularly when dealing with leaf veins, shadows, and areas where the background merges, their disease feature extraction capability is relatively weak, leading to a considerable number of false negatives and false positives, which directly impact the segmentation results and model performance.

Further comparative analysis shows that UNet and U2Net reduce the resolution of feature maps during the downsampling process, potentially limiting their capability to capture small-scale disease features. However, our MFBP-UNet, through the use of multi-scale convolutional kernels, has successfully extracted rich detail features while retaining global semantic information, enabling it to more effectively capture small-scale disease features.

The challenge of handling large-scale Slug images mainly stems from the fixed receptive field size of models like SegNet and HRNet, which may lead to the loss of context information when extracting large-scale disease features, affecting the model’s segmentation performance. Contrary to these models, MFBP-UNet, by incorporating the BATok-MLP module, manages to balance global and local information, thereby avoiding the loss of context information when dealing with large-scale disease features.

While models like SwinUNet and TransUNet have made improvements in certain respects, such as handling large-scale diseases, they still exhibit notable deficiencies in performance when dealing with complex backgrounds or tiny disease spots, resulting in less-than-ideal segmentation results.

After a deep comparison of these models, we discovered that our MFBP-UNet not only reduces the omission of disease spots but also makes significant improvements in edge detection, noticeably outperforming other models. This fully demonstrates the effectiveness of our strategy: by introducing the MFE and BATok-MLP modules and adopting weighted cross-entropy and Dice loss functions, we can extract and balance global and local feature information, reduce feature information loss, thereby significantly improving the model’s segmentation performance.

### 3.6. Model Performance Assessment: Ability to Deal with Different Types of Pear Leaf Diseases

To intuitively assess the performance of the MFBP-UNet model, we have utilized Grad-CAM [44] to visualize the areas of disease that the model focuses on. As illustrated in Table 6, we have chosen to compare this model with the top five networks based on Dice scores. The results show that MFBP-UNet can accurately concentrate on the disease areas across all types of plant diseases. Subsequently, we will demonstrate this specifically in the context of Rust, Leaf Curl, and Slug diseases.

First, in the detection of Rust disease, characterized by small, dense yellow spots on the leaves, MFBP-UNet successfully detects all disease areas, including those with smaller but denser spots.

Next, in the detection of Curl disease, which causes the edges of leaves to curl, our MFBP-UNet outperforms other networks in its ability to extract disease signs from the leaf edges.

Lastly, in the detection of Slug disease, which often forms large disease spots on the leaves, MFBP-UNet exhibits superior global information processing capability, enabling comprehensive detection of the diseased areas.

In conclusion, MFBP-UNet provides precise and detailed disease area segmentation, regardless of the type of disease or the size and distribution of the disease. This demonstrates its excellent performance in the task of pear leaf disease segmentation.

## 4. Discussion

In this study, we have successfully trained and validated an improved network based on the UNet architecture—the MFBP-UNet. This network is designed to handle pear leaf disease image segmentation tasks in natural environments and it has been thoroughly compared with various other segmentation networks. We found that each network architecture has its strengths and weaknesses in terms of performance on the task of pear leaf disease segmentation.

The UNet architecture is composed of two stages: contraction and expansion. Its strength lies in its ability to accurately capture and retain the contextual information of the image, but its inherent scaling operation might lead to the loss of information from small-scale targets. Therefore, based on this, we have optimized and designed new feature extraction modules—MFE and BATok-MLP.

The MFE module, by utilizing convolution kernels of various scales and types, effectively extracts detailed and semantic features. This allows it to understand multi-scale features, especially the detection ability of small targets, more deeply than the traditional UNet. To further improve the model’s robustness and enhance its ability to recognize disease features, we have introduced a frequency attention mechanism to replace traditional pooling operations. In the final stage of the model, we use the BATok-MLP module to reduce the complexity of the model. We also adopt a dynamic sparse attention mechanism to enhance the model’s use of global information, achieving a dynamic balance between global and local features.

To optimize the model, we used a combination of cross-entropy loss and Dice loss. This approach enables the model to effectively identify and locate pear leaf disease spots in complex environments. Compared with UNet and other advanced models, our optimization has significantly improved the overall performance and accuracy of the model.

We also noted that, while advanced segmentation networks like U2Net, TransUNet, SwinUNet perform excellently on some standard datasets, they did not perform as well in handling our task of pear leaf disease image segmentation. This could be due to issues like inconsistent shooting conditions, diversity of diseases, and scale changes in images taken in natural environments, or it could be due to insufficient dataset size. In addition, models with relatively fewer parameters, such as PSPNet, SegNet etc., might not have used pre-trained weights for training, which is crucial for small datasets. This could potentially lead to an inability to accurately capture the diverse features in our dataset.

Despite the excellent performance of our model in the current task, we recognize that there is much room for improvement in future research. For instance, we can further optimize the network architecture to better adapt to complex natural environments, or try to use more data augmentation and model fusion strategies to further enhance the robustness and accuracy of the model.

## 5. Conclusions

In this study, we focused on segmenting pear leaf disease images taken in natural environments and successfully developed an enhanced MFBP-UNet network. To effectively minimize the loss of small-scale target information and strengthen the integration capability of disease region edge details, we introduced innovations to the UNet structure. By integrating the MFE and BATok-Mlp modules, our network is equipped to capture and fuse features across different scales. The MFE module, utilizing multiple convolutional kernels, increases the sensitivity to smaller disease-affected regions. In contrast, the BATok-Mlp module, with its dynamic sparse attention mechanism and tokenized multi-layer perceptron components, strikes a balance between local and global information. Moreover, by combining cross-entropy loss with Dice loss, we have enhanced the accuracy in identifying and locating pear leaf lesions.

Compared to the original UNet, our modified network model shows superior segmentation performance. Specifically, our method achieved scores of 86.15%, 93.53%, 90.89%, and 0.922 on performance metrics such as MIoU, MP, MPA, and Dice, respectively.

While our method is effective at segmenting pear leaf lesions, the network model is notably larger. To further optimize the model’s performance and efficiency in the future, we plan to continuously enrich the dataset on pear leaf diseases, explore new strategies to simplify the model, and integrate our solution into the latest disease detection platforms. This approach aims to achieve higher segmentation precision and efficiency, making it more suitable for real-world production applications. Code is available at https://github.com/Lancelot-wy/MFBP-UNet-A-Network-for-Pear-Leaf-Disease-Segmentation-in-Natural-Agricultural-Environments (accessed on 23 August 2023).

## Figures and Tables

**Table 1:** Number and proportion of pear leaf disease images.

Categories	Example	Number (before)	Number (after)	Proportion (after)
Rust	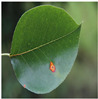	838	1676	35.11
Slug	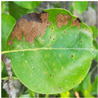	421	1684	35.28
Curl	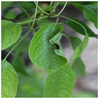	157	1413	29.60

**Table 2:** Hardware and software parameters.

**Hardware environment**	CPU	Intel(R)Xeon(R)Platinum 8350C CPU @ 2.60 GHz
	GPU	RTX 3090*2
	RAM	64 GB
	Video Memopry	24 GB
**Software environment**	OS	Ubuntu 18.04.5 LTS
	CUDA Toolkit	V11.1
	CUDNN	V8.0.4
	Python	3.8.8
	torch	1.8.1
	torchvision	0.9.1

**Table 3:** Experimental settings.

Parameters	Value
Size of input images	512 × 512 pixels
Batch size	8
Maximum learning rate	0.0001
Minimum learning rate	0.000001
Optimizer	Adam
Momentum	0.9
Number of iterations	100 epochs

**Table 4:** Comparison of the main performance of different methods.

Model	MIoU	MPrecision	MPA	Dice	FPS	Parameters
DeepLabV3	83.39%	90.53%	90.82%	0.902	5.709	58.626628 M
HRNet	81.28%	89.20%	89.33%	0.889	5.625	51.949141 M
TransUNet	62.47%	68.28%	83.27%	0.828	5.620	67.865764 M
UNet	80.40%	87.74%	89.81%	0.848	5.991	17.263042 M
PSPNet	82.06%	89.46%	90.11%	0.859	7.430	49.068488 M
U2Net	83.68%	92.08%	89.65%	0.905	4.142	44.037052 M
UNet++	74.46%	88.63%	89.85%	0.851	5.873	26.905685 M
FCN	83.21%	90.55%	90.55%	0.904	6.143	9.641661 M
Lraspp	76.06%	85.57%	85.67%	0.847	7.640	3.218648 M
SegNet	77.18%	86.01%	86.78%	0.871	5.321	29.445316 M
SwinUNet	61.84%	72.69%	75.70%	0.681	6.187	41.393124 M
MFBP-UNet	86.15%	93.53%	90.89%	0.922	4.603	22.076297 M

**Table 5:** The effect of running various methods.

	Rust	Curl	Slug
Disease image	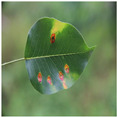	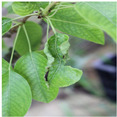	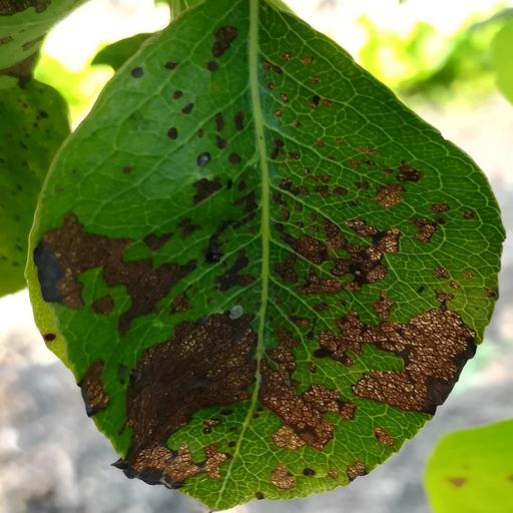
Ground truth	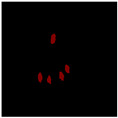	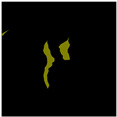	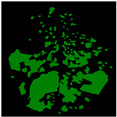
DeepLabV3	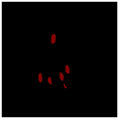	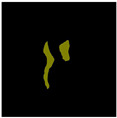	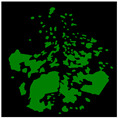
HRNet	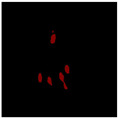	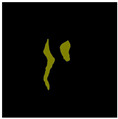	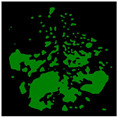
TransUNet	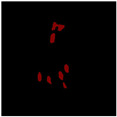	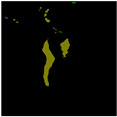	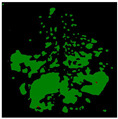
UNet	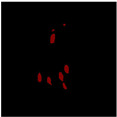	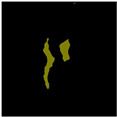	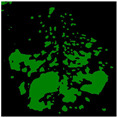
PSPNet	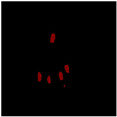	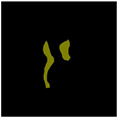	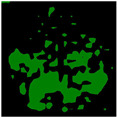
U2Net	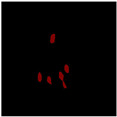	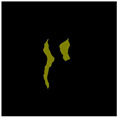	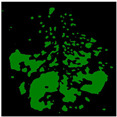
UNet++	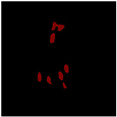	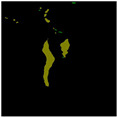	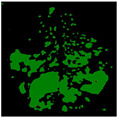
FCN	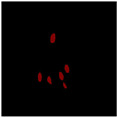	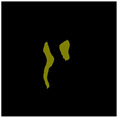	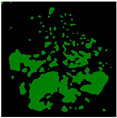
Lraspp	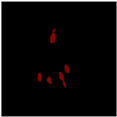	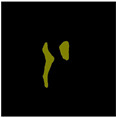	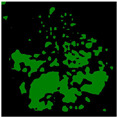
SegNet	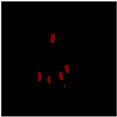	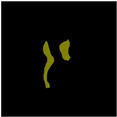	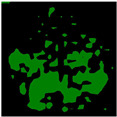
SwinUNet	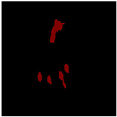	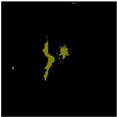	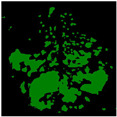
MFBP-UNet	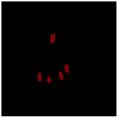	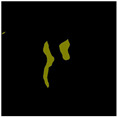	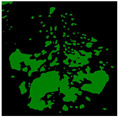

**Table 6:** Heatmaps of different methods using Grad-CAM.

	Rust	Curl	Slug
Disease image	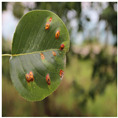	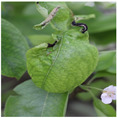	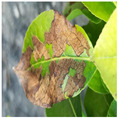
DeepLabV3	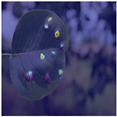	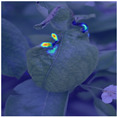	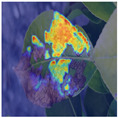
HRNet	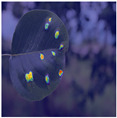	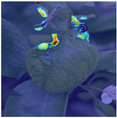	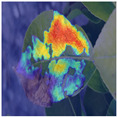
FCN	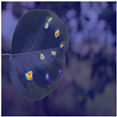	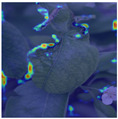	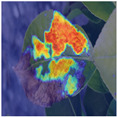
U2Net	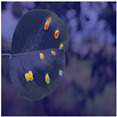	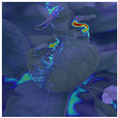	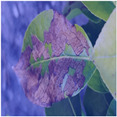
MFBP-UNet	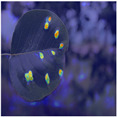	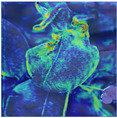	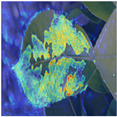

## Data Availability

Not applicable.

## References

[1] Zhang S., Xie Z. (2019). Current status, trends, main problems and the suggestions on development of pear industry in China. J. Fruit Sci..

[2] Fenu G., Malloci F.M. (2021). Using multioutput learning to diagnose plant disease and stress severity. Complexity.

[3] Zhao Y., Tian Y., Wang L., Geng G., Zhao W., Hu B., Zhao Y. (2019). Fire blight disease, a fast-approaching threat to apple and pear production in China. J. Integr. Agric..

[4] Varoquaux G., Cheplygina V. (2022). Machine learning for medical imaging: Methodological failures and recommendations for the future. NPJ Digit. Med..

[5] Zheng Y., Song Q., Liu J., Song Q., Yue Q. (2020). Research on motion pattern recognition of exoskeleton robot based on multimodal machine learning model. Neural Comput. Appl..

[6] Saha D., Manickavasagan A. (2021). Machine learning techniques for analysis of hyperspectral images to determine quality of food products: A review. Curr. Res. Food Sci..

[7] Tahseen Ali A., Abdullah H.S., Fadhil M.N. (2021). Voice recognition system using machine learning techniques. Mater. Today Proc..

[8] Sujatha R., Chatterjee J.M., Jhanjhi N., Brohi S.N. (2021). Performance of deep learning vs machine learning in plant leaf disease detection. Microprocess. Microsyst..

[9] Poornima S., Kavitha S., Mohanavalli S., Sripriya N. (2019). Detection and classification of diseases in plants using image processing and machine learning techniques. AIP Conf. Proc..

[10] Ma J., Du K., Zheng F., Zhang L., Sun Z. (2019). A segmentation method for processing greenhouse vegetable foliar disease symptom images. Inf. Process. Agric..

[11] Jothiaruna N., Sundar K.J.A., Karthikeyan B. (2019). A segmentation method for disease spot images incorporating chrominance in comprehensive color feature and region growing. Comput. Electron. Agric..

[12] Dutta K., Talukdar D., Bora S.S. (2022). Segmentation of unhealthy leaves in cruciferous crops for early disease detection using vegetative indices and Otsu thresholding of aerial images. Measurement.

[13] Dhanachandra N., Manglem K., Chanu Y.J. (2015). Image segmentation using K-means clustering algorithm and subtractive clustering algorithm. Procedia Comput. Sci..

[14] Kartikeyan P., Shrivastava G. (2021). Review on emerging trends in detection of plant diseases using image processing with machine learning. Int. J. Comput. Appl..

[15] Ale L., Sheta A., Li L., Wang Y., Zhang N. Deep learning based plant disease detection for smart agriculture. Proceedings of the 2019 IEEE Globecom Workshops (GC Wkshps).

[16] Bock C.H., Barbedo J.G., Del Ponte E.M., Bohnenkamp D., Mahlein A.K. (2020). From visual estimates to fully automated sensor-based measurements of plant disease severity: Status and challenges for improving accuracy. Phytopathol. Res..

[17] Tassis L.M., Tozzi de Souza J.E., Krohling R.A. (2021). A deep learning approach combining instance and semantic segmentation to identify diseases and pests of coffee leaves from in-field images. Comput. Electron. Agric..

[18] He K., Gkioxari G., Dollár P., Girshick R. Mask r-cnn. Proceedings of the IEEE international Conference on Computer Vision.

[19] Ronneberger O., Fischer P., Brox T. (2015). U-net: Convolutional networks for biomedical image segmentation. Medical Image Computing and Computer-Assisted Intervention—MICCAI 2015, Proceedings of the 18th International Conference, Munich, Germany, 5–9 October 2015.

[20] Zhao H., Shi J., Qi X., Wang X., Jia J. Pyramid scene parsing network. Proceedings of the IEEE Conference on Computer Vision and Pattern Recognition.

[21] He K., Zhang X., Ren S., Sun J. Deep residual learning for image recognition. Proceedings of the IEEE Conference on Computer Vision and Pattern Recognition.

[22] Patil M.A., M M. (2022). Enhanced radial basis function neural network for tomato plant disease leaf image segmentation. Ecol. Inform..

[23] Wang R., Cheng M., Yuan H., Zhu J., Wang Q., Cai Z. (2022). An Improved DeepLab v3+ Deep Learning Network Applied to the Segmentation of Grape Leaf Black Rot Spots. Front. Plant Sci..

[24] Chen L.C., Zhu Y., Papandreou G., Schroff F., Adam H. Encoder-decoder with atrous separable convolution for semantic image segmentation. Proceedings of the European Conference on Computer Vision (ECCV).

[25] Zhang S., Zhang C. (2023). Modified U-Net for plant diseased leaf image segmentation. Comput. Electron. Agric..

[26] Li K., Zhang L., Li B., Li S., Ma J. (2022). Attention-optimized DeepLab V3+ for automatic estimation of cucumber disease severity. Plant Methods.

[27] Fenu G., Malloci F.M. (2021). DiaMOS plant: A dataset for diagnosis and monitoring plant disease. Agronomy.

[28] Radford A., Kim J.W., Hallacy C., Ramesh A., Goh G., Agarwal S., Sastry G., Askell A., Mishkin P., Clark J. Learning transferable visual models from natural language supervision. Proceedings of the 38th International Conference on Machine Learning—PMLR.

[29] Valanarasu J.M.J., Patel V.M. (2022). Unext: Mlp-based rapid medical image segmentation network. Medical Image Computing and Computer Assisted Intervention – MICCAI 2022, Proceedings of the 25th International Conference, Singapore, 18–22 September 2022.

[30] Zhu L., Wang X., Ke Z., Zhang W., Lau R.W. BiFormer: Vision Transformer with Bi-Level Routing Attention. Proceedings of the IEEE/CVF Conference on Computer Vision and Pattern Recognition.

[31] Glorot X., Bordes A., Bengio Y. Deep sparse rectifier neural networks. Proceedings of the Fourteenth International Conference on Artificial Intelligence and Statistics.

[32] Everingham M., Van Gool L., Williams C.K., Winn J., Zisserman A. (2010). The pascal visual object classes (voc) challenge. Int. J. Comput. Vis..

[33] Dice L.R. (1945). Measures of the amount of ecologic association between species. Ecology.

[34] Tang Z., He X., Zhou G., Chen A., Wang Y., Li L., Hu Y. (2023). A Precise Image-Based Tomato Leaf Disease Detection Approach Using PLPNet. Plant Phenomics.

[35] Zhou Z., Rahman Siddiquee M.M., Tajbakhsh N., Liang J. (2018). Unet++: A nested u-net architecture for medical image segmentation. Deep Learning in Medical Image Analysis and Multimodal Learning for Clinical Decision Support, Proceedings of the 4th International Workshop, DLMIA 2018, and 8th International Workshop, ML-CDS 2018, Held in Conjunction with MICCAI 2018, Granada, Spain, 20 September 2018.

[36] Qin X., Zhang Z., Huang C., Dehghan M., Zaiane O.R., Jagersand M. (2020). U2-Net: Going deeper with nested U-structure for salient object detection. Pattern Recognit..

[37] Chen J., Lu Y., Yu Q., Luo X., Adeli E., Wang Y., Lu L., Yuille A.L., Zhou Y. (2021). Transunet: Transformers make strong encoders for medical image segmentation. arXiv.

[38] Cao H., Wang Y., Chen J., Jiang D., Zhang X., Tian Q., Wang M. Swin-unet: Unet-like pure transformer for medical image segmentation. Proceedings of the European Conference on Computer Vision.

[39] Long J., Shelhamer E., Darrell T. Fully convolutional networks for semantic segmentation. Proceedings of the IEEE Conference on Computer Vision and Pattern Recognition.

[40] Chen L.C., Papandreou G., Schroff F., Adam H. (2017). Rethinking atrous convolution for semantic image segmentation. arXiv.

[41] Sun K., Xiao B., Liu D., Wang J. Deep high-resolution representation learning for human pose estimation. Proceedings of the IEEE/CVF Conference on Computer Vision and Pattern Recognition.

[42] Howard A., Sandler M., Chu G., Chen L.C., Chen B., Tan M., Wang W., Zhu Y., Pang R., Vasudevan V. Searching for mobilenetv3. Proceedings of the IEEE/CVF International Conference on Computer Vision.

[43] Badrinarayanan V., Kendall A., Cipolla R. (2017). Segnet: A deep convolutional encoder-decoder architecture for image segmentation. IEEE Trans. Pattern Anal. Mach. Intell..

[44] Selvaraju R.R., Cogswell M., Das A., Vedantam R., Parikh D., Batra D. Grad-cam: Visual explanations from deep networks via gradient-based localization. Proceedings of the IEEE International Conference on Computer Vision.

